# Occupational musculoskeletal and mental disorders as the most frequent associations to worker’s sickness absence: A 10-year cohort study

**DOI:** 10.1186/1756-0500-5-229

**Published:** 2012-05-11

**Authors:** Antonio Carlos Zechinatti, João Carlos Belloti, Vinícius Ynoe de Moraes, Walter Manna Albertoni

**Affiliations:** 1 Department of Orthopedics and Traumatology, Universidade Federal de São Paulo – Escola Paulista de Medicina, Rua Borges Lagoa, 783 - 5th Floor, São Paulo, Brazil; 2 Divisão de Perícia Médica, Serviço Especializado em Saúde e Medicina do Trabalho - SESMT, Rua Mirassol, 315, Vila Clementino, São Paulo, SP, 04020-060, Brazil

**Keywords:** Occupational diseases, Health personnel, Absenteeism, International classification of diseases, Brazil

## Abstract

**Background:**

Sickness absence (SA) is a complex phenomenon influenced by the health of the worker and socio-economic factors. An epidemiological study of SA has never been conducted for Brazilian university workers. This study aimed to determine the main diseases that are associated with SA and find out the average length of SA duration, and its variation among different staff members and between sexes over the 10-year study period.

**Methods:**

We identified the main diseases responsible for SA in workers at a Brazilian federal university (UNIFESP) from January 1998 to August 2008 and grouped them according to the International Classification of Diseases (ICD10). Independent researchers assessed data collected from expert reports of the university Worker’s Health Division.

**Results:**

During the period of our study, 1176 workers experienced sickness absence. After evaluating 7579 consultations, ICD10 distribution showed that musculoskeletal and connective tissue disorders (“M” axis) and mental and behavioral diseases (“F” axis) were the most important causes of SA, occurring in 47.3% (IC 95%; 44.15-49.8) of workers aged 46.2 (SD 10.1) years. Female workers represented 78.1% (IC 95%; 76-80.7) of all workers with SA, but men had higher proportional rates (Chi-square; p = 0.044). Longer SA periods were observed for illnesses related to neoplasms and infectious diseases.

**Conclusions:**

Musculoskeletal and connective tissue disorders and mental and behavioral diseases were the most frequent cause of sickness absence. Men had an increased frequency of SA, and neoplasms and infectious disorders were associated with longer absences. Mostly, these are occupational disorders. A preventative research-focused agenda is desirable for a more accurate depiction of this population in the scope of policy-making. Our results for SA in Brazilian workers correspond with those of other studies worldwide.

## Background

Absenteeism or sickness absence (SA) is a worldwide concern due to the economic consequences for the employer, for government or profitable institutions, and for the workers themselves. A broad spectrum of factors influences SA, including demographics, health systems, health status, and preventative occupational actions. By promoting prompt detection mechanisms, workers at risk can be readily identified and treated [1-3].

Epidemiological patterns of absenteeism have recently shifted, showing an increase in psychosocial disorders in contrast to a decreasing, but still frequent, incidence of musculoskeletal and connective tissue diseases, especially in countries of the European Union. This shift in the epidemiological profile of SA raises concern for public and private institutions, since SA significantly impacts budgets and contributes to the deterioration of employee health [3-9]. Besides of its established evidence - recognized mostly in European countries, this shift was not recognized or studied in Brazil.

Among the expected benefits of a recent Brazilian federal law, employees have the right to leave work for health reasons that is determined necessary by a medical expert, without prejudice regarding their entitled wages [10]. Correlation of variables such as age, gender, job function, and disease (ICD10 - World Health Organization, 1998) [11] sets the parameters to evaluate the causes of absences, and whether or not these causes are characterized as occupational diseases. Federal law stipulates that disability retirement must be preceded by a medical leave of absence for a period not exceeding 24 months. Workers must retire if their licenses expire or if they are unable to return to their original jobs and cannot be reassigned to a new position [10].

In this study we evaluated causes of absenteeism within a population of workers based at a federal university and focused our analysis on assessments provided by the workers’ health division (WHD), which granted sick-leave requests upon the recommendation of a medical expert. The study focus in the recognition of a Brazilian “primer” regarding to SA motivations and its epidemiological profile. This research is an initiative that could encourage a rationale for an evidence-based national agenda [12], which is relevant for policy-makers, physicians and workers.

The aim of this study was not only to determine the main diseases that cause SA, but also to construct an epidemiological profile of these workers, recognize the relationship between SA and job function and the outcome of medical examinations performed over the 10-year period of the study.

## Methods

### Study design and population characteristics

We conducted a historical cohort study of the employees of a Brazilian federal university over an assessment period of 10 consecutive years (1998-2008). This public university employs 5000 workers, including nursing assistants, personnel assistants, engineers, physicians, teachers, and manual workers.

This study is based upon a series of consultations conducted by a specialized health service for the workers (the WHD). This health service provides workers with access to a multidisciplinary medical team, coordinated by physicians who are responsible for the final decision on the worker’s health status. When out due to SA, visiting WHD for periodical consultations is compulsory. The WHD team is the foremost responsible for deciding the employee destiny after sickness absence, occasionally aided by an external expert.

Data analysis for the total population that used this service during the period from 1998 to 2008 was based on medical (medical records) and legal documentation (Human Resources registries), which gave us access to the following epidemiological data: age, occupation, title, education level, the disease that motivated the consultation, the health reason that prompted the worker’s absence, and the stratification of these illnesses according to ICD10. We also collected data relating to the SA, including work time at the university, work time prior to SA, and time off due to sickness. These data were extracted manually from the WHD of the university by two researchers unrelated to this study and were checked against an electronic database from the Human Resources (HR) division of the university to validate their accuracy. No self-reported data were obtained in this study.

The population included in this study consisted of employees who had been admitted to the WHD and who were subsequently absent from their work activity for a minimum period of 30 calendar days due to sickness. These criteria led us to the inclusion of long-term SA, which is the exact of the population that requests Brazilian government insurances. A team experienced in occupational medicine should have assessed this information, and during the period of our study, they have strictly followed Brazilian law consensus, as exposed above. We excluded workers with SA that could possibly be motivated by factors other than health issues (e.g. concomitant judicial issues) and employees whose data were inconsistent between the WHD and the HR division. The local ethics committee (Comite de Ética em Pesquisa – UNIFESP/EPM) approved this study.

## Study variables

The goal of this study was to verify the diagnoses that lead to the absenteeism of workers, to calculate the average time of SA according to ICD10 *(appendix*1*and*2*)*, and to determine the correlation of SA with gender and professional activities. We have grouped the data according to professional activities.

## Statistical methods

For categorical variables, descriptive statistics were used with absolute and relative frequencies to delineate the sample. For numerical variables, continuous, descriptive statistics were applied to show the distribution by utilizing measures of central tendency and dispersion, with alpha at 0.05. When choosing the group of statistical test that would be used for statistical inference, we applied the Kolmogorov-Smirnov normality test with the purpose of checking the normal distribution of the data. We found that our data were normally distributed. We used the *t*-test for the comparison of means with significance levels of 95%. We used SAS software for statistical analysis.

## Results

### Epidemiological aspects

We analyzed data from a total of 7579 medical examinations administered to 1176 participants between 1998 and 2008. The average age at the time of the participant’s first consultation with the WHD was 46.2 years, and the average number of consultations per participant was 6.4.

Of the participants in this study, 78.4% were women. It should be noted that the university employees are mostly women (80.1%). Applying analysis for difference of proportions, we observed that men filed significantly more sick-leave reports than women (Chi-square test; p = 0.044). The average sick leave per worker was 243.8 days Tables 1 and 2 summarize the epidemiological aspects of the analyzed population.

**Table 1:** Age of workers and number of workers’ health division (WHD) reports analyzed in this study

**Variables**	**Median**	**Mean**	**SD**
Age (years)	47 (19-69)	46.2	10.1
Number of WHD reports (n)	3 (1-49)	6.4	7.2

**Table 2:** Gender distribution of workers: SA workers vs. total university personnel

**Gender**	**SA workers *n (%)***	**Total population *n (%)***
Female	922 (78.4)	6130 (80.9)
Male	254 (21.6)	1449 (19.1)
	1176 (100)	7579 (100)

**Chi Square Test, *****p = 0.044.***

### Major causes of absenteeism among the workers

Musculoskeletal and connective tissue disorders (ICD10 “M” axis) caused the majority of SA during the period of this study, followed by mental health and behavioral disorders (ICD10 “F” axis) (Table 3). The distribution of these diagnoses between males and females is shown in Table 4. According to ICD10 classification, diagnoses fell into the following subgroups: severe depressive episodes without psychotic symptoms (F32.2), essential (primary) hypertension (I10), convalescence following surgery (Z54.0), shoulder lesions (M75), moderate depressive episodes (F32.1), dorsalgia (M54), adjustment disorders (F43.2), chronic ischemic heart disease (I25), recurrent depressive disorders, and current episodes of severe depression without psychotic symptoms (F33.2) (Table 3 and 4).

**Table 3:** Gender distribution of participants according to ICD10 classification

**ICD10 axis**	**Male n (%)**	**Female n (%)**	**Total**
M	278 (19.2)	1514 (24.7)	1792 (23.6)
F	246 (17)	1502 (24.5)	1748 (23.1)
S	229 (15.8)	575 (9.4)	804 (10.6)
I	182 (12.6)	470 (7.7)	652 (8.6)
Z	93 (6.4)	375 (6.1)	468 (6.2)
C	85 (5.9)	266 (4.3)	351 (4.6)
G	42 (2.9)	167 (2.7)	209 (2.8)
H	27 (1.9)	144 (2.3)	171 (2.3)
J	10 (0.7)	159 (2.6)	169 (2.2)
D	0 (0)	163 (2.7)	163 (2.2)
K	23 (1.6)	134 (2.2)	157 (2.1)
N	25 (1.7)	117 (1.9)	142 (1.9)
E	40 (2.8)	100 (1.6)	140 (1.8)
B	57 (3.9)	67 (1.1)	124 (1.6)
D	0 (0)	120 (2)	120 (1.6)
R	20 (1.4)	80 (1.3)	100 (1.3)
L	26 (1.8)	71 (1.2)	97 (1.3)
D	20 (1.4)	68 (1.1)	88 (1.2)
T	44 [12]	26 (0.4)	70 (0.9)
Q	0 (0)	5 (0.1)	5 (0.1)
Y	0 (0)	4 (0.1)	4 (0.1)
K	2 (0.1)	0 (0)	2 (0)
P	0 (0)	2 (0)	2 (0)
W	0 (0)	1 (0)	1 (0)
TOTAL	1449 (19.1)	6130 (80.9)	7579 (100.0)

**Table 4:** Disease specific distribution, according to ICD10 diagnosis

**ICD10**	**N (%)**
F32.2	456 (6)
I10	207 (2.7)
Z54.0	199 (2.6)
M75	141 (1.9)
F32.1	139 (1.8)
M54	121 (1.6)
F43.2	98 (1.3)
I25	97 (1.3)
F32	94 (1.3)
F33.2	89 (1.2)

### The length of sick leave according to ICD10 diagnosis

Neoplasms (“C” axis) caused the longest sick leave periods, followed by certain infectious and parasitic diseases (“B” axis) and mental and behavioral disorders (“F” axis). The lowest average times of leave were in the groups of respiratory diseases (“J” axis) and certain conditions originating in the perinatal period (“P” axis) (Table 5).

**Table 5:** Mean length of sick leave according to ICD10 diagnosis

**ICD10**	**Median (days; min-max)**	**Mean**	**SD**
C	60 (1-586)	58.7	44.6
B	42 (1-187)	49.0	36.5
F	35 (1-366)	42.5	31.2
E	31 (1-221)	41.9	38.2
G	32 (1-212)	41.3	34.8
M	34 (1-436)	41.2	35.8
Y	32 (5-95)	41.0	38.3
I	34 (1-362)	41.0	36.3
T	32 (1-113)	37.7	30.2
Q	15 (5-99)	37.4	42.0
K	30 (1-111)	34.9	31.2
S	30 (1-137)	32.5	25.7
O	19 (1-751)	30.3	62.0
D	20 (1-99)	29.4	27.0
A	22 (1-95)	27.2	27.2
Z	21 (1-285)	27.0	28.5
R	8.5 (1-285)	22.1	29.9
H	10 (1-92)	20.5	22.0
L	14 (1-69)	20.0	18.1
W	18 (18)	18.0	.
N	11 (1-116)	17.8	20.6
P	16.5 (1-132)	16.5	21.9
J	8 (1-76)	15.6	16.8

### Time away from work according to labor function

Nursing professionals had the largest mean number of lost workdays (132.9 days), followed by administrative workers (43.1 days). Pharmacy workers were absent for the fewest number of days (2.9 days), followed by teachers (4.34 days) (Table 6). Based on analysis of workers who used the services of the WHD, nursing assistants requested the most examinations (46%), followed by administrative workers (14%), both of which were predominantly women (Table 7), although it should be noted that the majority of participants in all functional categories except maintenance and engineering were women.

**Table 6:** Average length of sick leave according to functional group

**Functional group**	**Cases (n)**	**Median (days; range)**	**Mean**	**SD**
Other Operational	96	122.5 (1-1406)	312.7	374.5
Operational Center of Nutrition and Dietetics	74	111.5 (1-1241)	312.3	370.3
Maintenance Engineering	67	104 (1-1370)	277.7	358.5
Nursing Care	539	84 (1-2009)	246.7	337.3
Administrative	171	98 (1-1558)	229	296.2
Pharmacy	13	128 (2-793)	223.1	270.2
Healthcare Laboratory	94	89 (1-1160)	212.6	275.9
Professor	23	105 (1-758)	189	224.3
Other Assistance	43	73 (5-776)	174.1	216.6
Medical Care	56	52.5 (3-1761)	146.5	278.2

**Table 7:** Work occupation and gender of participants who used the WHD services

**Occupation**	**Male n (%)**	**Female n (%)**	**Total**
Nursing Care	56 (10.4)	483 (89.6)	539 (45.8)
Administrative	39 (228)	132 (77.2)	171 (14.5)
Other, Operational	39 (40.6)	57 (59.4)	96 (8.2)
Healthcare, Laboratory	27 (28.7)	67 (71.3)	94 (8.0)
Operational, Center of Nutrition and Dietetics	4 (5.4)	70 (94.6)	74 (6.3)
Medical Care assistant	22 (39.3)	34 (60.7)	56 (4.8)
Maintenance and Engineering	47 (70.1)	20 (29.9)	67 (5.7)
Other, Assistance	13 (30.2)	30 (69.8)	43 (3.7)
Professor	6 (26.1)	17 (73.9)	23 (2.0)
Pharmacy	1 (7.7)	12 (92.3)	13 (1.1)
Total	254 (21.6)	922 (78.4)	1176 (100.0)

### Results of the workers’ health division records

After analyzing the reports of medical experts in the WHD, we found that the most common outcome was a return to previous activities after the sick leave period (Table 8).

**Table 8:** Outcomes of WHD evaluation

**Outcome**	**Male, n (%)**	**Female, n (%)**	**Total, n (%)**
Returned to their normal work	160 (22.4)	553 (77.6)	713 (60.7)
Retirement	67 (18.8)	290 (81.2)	357 (30.4)
Death	19 (28.4)	48 (71.6)	67 (5.7)
Exoneration	7 (23.3)	23 (76.7)	30 (2.5)
Redistribution	1 (11.1)	8 (88.9)	9 (0.8)
TOTAL	254 (21.6)	922 (78.4)	1176 (100.0)

## Discussion

Because our data came from a population based at a university that specializes in the health sciences, such as medicine, nursing, and biomedical sciences, we presumed that we would find a high number of medical workers developing SA. Regarding the causes of SA, our results correspond well with those of previous studies [13-18], which identified musculoskeletal and connective tissue disorders and mental illness as the main causes of absenteeism [18,19].

The Brazilian law system stipulates a maximum length of 2 years for sickness absence; after this period of time the worker should be relocated or retired. Implementing a clear time period for insurance support possibly avoids insurance misuse. In this regard Brazil differs from more socially focused insurance systems, such as the Swedish system, in which it is possible that the excessive protection of SA workers threatens the welfare state [19,20].

Apart from the limitations on sick leave imposed by the Brazilian government, few comprehensive studies of absenteeism in Brazil have been performed [14-16,18,21,22], and most of these studies targeted specific professional groups or sectors [23]. Our data suggest that the pattern of disease associated with absenteeism resembles that in other countries where comprehensive data are available [19,24-26]. Our large sample group, comprised of 5000 workers, and the wide scope of our data due to the diversity of workers’ skills, strengthen this conclusion. However, we must consider certain limitations of our study, especially regarding the nature of our data collection and study design. We analyzed a specific population (university personnel/São Paulo Metropolitan area) and part of the data was obtained retrospectively.

Our research is important since it reveals some aspects of Brazilian’s worker health and our society. Musculoskeletal conditions – mostly occupational - and psychological disorders emerged as the most relevant causes incurring at Sickness absence in our population and we believe that our sample most likely reflects the general Brazilian working population because we included a great variety of working categories and we included data from a large (representative) sample. This fact is relevant, since besides the differences among different country’s, culture and population income, we believe that working diseases might have a more universal characteristic, which sounds similar to worldwide studies.

We believe that facing these conditions and proposing preventative programs for dealing with these conditions is a cornerstone action. This panorama has led us to the development of a robust research agenda, which is focused in the recognition of avoidable risk factors and clinical studies for determining the effectiveness of alternative interventional actions.

The main outcomes of WHD expert reports on workers granted sick leave included retirement (30.4%), return to original activities (60.7%), and death (5.7%). Our results regarding the length of SA are also consistent with findings of this previous study, in which tumors were the main diagnosis leading to a long period of SA [27].

## Conclusions

Musculoskeletal and connective tissue disorders and mental and behavioral diseases were the most frequent cause of sickness absence. Men had an increased frequency of SA, and neoplasms and infectious disorders were associated with longer absences. Mostly, these are occupational disorders. A preventative research-focused agenda is desirable for a more accurate depiction of this population in the scope of policy-making. Our results for SA in Brazilian workers correspond with those of other studies worldwide.

## Appendix 1

### ICD10 grouping

M -Diseases of the musculoskeletal system and connective tissue

F Mental and behavioural disorders

S Injury, poisoning and certain other consequences of external causes

I Diseases of the circulatory system

Z Factors influencing health status and contact with health services

C Neoplasms

G Diseases of the nervous system

H Diseases of the eye and adnexa, Diseases of the ear and mastoid process

J Diseases of the respiratory system

N Diseases of the genitourinary system

E Endocrine, nutritional and metabolic diseases

B Certain infectious and parasitic diseases

D Diseases of the blood and blood-forming organs and certain disorders involving the immune mechanism,

R Symptoms, signs and abnormal clinical and laboratory findings, not elsewhere classified

L Diseases of the skin and subcutaneous tissue

D Diseases of the blood and blood-forming organs and certain disorders involving the immune mechanism

T Injury, poisoning and certain other consequences of external causes

Q Congenital malformations, deformations and chromosomal abnormalities

Y External causes of morbidity and mortality

K Diseases of the digestive system

P Certain conditions originating in the perinatal period

## Appendix 2

## Disease specific ICD10 distribution

F32.2 Severe depressive episode without psychotic symptoms

I10 Essential (primary) hypertension

Z54.0 Convalescence following surgery

M75 Shoulder Lesions

F32.1 Moderate Depressive episode

M54 Dorsalgia

F43.2 Adaptation Reaction

I25 Chronic ischaemic heart disease

F32 Depressive Episode

F33.2 Recurrent depressive disorder, current episode severe without psychotic symptoms

## Competing interests

The authors declare that they have no competing interests.

## Authors’ contributions

ACZ carried out manuscript design and data collection. JCB conceived the study, and participated in its design and coordination and helped to draft the manuscript. VYM helped to draft the manuscript and performed part of the statistical analysis. WMA reviewed the final manuscript. All authors read and approved the final manuscript.
